# The Wise Women of Nursing

**Published:** 1919-11-22

**Authors:** 


### THE TRAINED NURSES' PAGES.

### THE WISE WOMEN OF NURSING.

After-war conditions have produced a state of general restlessness and upset in all classes of people. There never was a time when experience and wisdom in individuals possessed a greater value and power for good than they possess to-day. Sir Arthur Stanley's letter puts on record the volume of excellent work which has been done for and by nurses, mainly since the College of Nursing was incorporated. This record is a remarkable exhibition of what can be accomplished under continuous difficulties by wise counsel and willing workers. The intelligence of the whole nursing profession, as represented by its individual members, must now surely realise, that the future of every nurse will be materially aided and improved through the College, which has earned their confidence and should possess that of every intelligent trained nurse, in full measure. This being so, a great field for personal service on behalf of their profession and fellow workers lies open for cultivation by the Wise Women amongst them. We should like to get into touch with every one of them who has ideas and the will to work for her fellows, because we see the way whereby they can collectively and individually accomplish a great work for their profession, should they be willing and anxious to devote such leisure as they have to its accomplishment. Every one anxious to lend a hand is invited to send name, address and qualifications to the editor.

In the past, nurses have lacked leisure, ample relaxation and wholesome amusement. The Nurses' Registration (No. 2) Bill, prepared by Dr. Addison, the Minister of Health, which was introduced on November 6, passed its second reading without opposition on the 18th instant. Before it was printed Mrs. Fenwick, at the meeting she addressed on the 7th instant, gave her audience to understand that the State Registration Bill Dr. Addison had introduced was that of the Central Committee. Will one or some of the Wise Women of the Nursing profession use a little of their leisure, and by close study of Dr. Addison's Nurses' Registration (No. 2) Bill (see page 175 this week's The Hospital) endeavour to discover the Bill of the Central Committee within the four corners of the Bill which passed its second reading in the House of Commons on Tuesday evening last? We shall welcome the result of their sporting efforts to find the impossible.

It is time the whole nursing profession awakes, gets to business and ceases to be troubled by the innumerable hares which have been started to mislead the nurses and further the ambitions of a very few people who, almost without exception, have had nothing to do with the training of nurses for decades of years. Registration is safe in the hands of Dr. Addison and the Government, and trained nurses who are wise will forthwith devote their whole energies to recruiting for the College Register and the building up of everything which can help the College to help all trained nurses to the uttermost. The first step is for each to recruit at least *one new member* for the College Register.

